# HtrA chaperone activity contributes to host cell binding in *Campylobacter jejuni*

**DOI:** 10.1186/1757-4749-3-13

**Published:** 2011-09-22

**Authors:** Kristoffer T Bæk, Christina S Vegge, Lone Brøndsted

**Affiliations:** 1 Department of Veterinary Disease Biology, University Copenhagen, Stigbøjlen 4, DK-1870 Frederiksberg C, Denmark

**Keywords:** HtrA, chaperone, protease, *Campylobacter jejuni*, INT-407, phagocytosis, virulence

## Abstract

**Background:**

Acute gastroenteritis caused by the food-borne pathogen *Campylobacter jejuni *is associated with attachment of bacteria to the intestinal epithelium and subsequent invasion of epithelial cells. In *C. jejuni*, the periplasmic protein HtrA is required for efficient binding to epithelial cells. HtrA has both protease and chaperone activity, and is important for virulence of several bacterial pathogens.

**Results:**

The aim of this study was to determine the role of the dual activities of HtrA in host cell interaction of *C. jejuni *by comparing an *htrA *mutant lacking protease activity, but retaining chaperone activity, with a Δ*htrA *mutant and the wild type strain. Binding of *C*. *jejuni *to both epithelial cells and macrophages was facilitated mainly by HtrA chaperone activity that may be involved in folding of outer membrane adhesins. In contrast, HtrA protease activity played only a minor role in interaction with host cells.

**Conclusion:**

We show that HtrA protease and chaperone activities contribute differently to *C. jejuni*'s interaction with mammalian host cells, with the chaperone activity playing the major role in host cell binding.

## Background

The enteric pathogen *Campylobacter jejuni *is a frequent cause of bacterial food-borne infections worldwide [1]. Acute gastroenteritis caused by *C. jejuni *is characterized by watery or bloody diarrhea, abdominal pain, fever, and malaise. While these symptoms typically last 3 - 7 days, serious complications may follow such as the acute autoimmune disease Guillan Barré Syndrome, affecting the peripheral nervous system. To cause disease in humans, *C*. *jejuni *must penetrate the mucus layer of the gastrointestinal epithelium and interact with the underlying epithelial cells [2]. The importance of epithelial cell invasion in disease has been demonstrated in infected humans and animals [3,4], and is emphasized by studies showing that *C. jejuni *mutants attenuated for virulence in animal models are less capable of invading intestinal epithelial cells *in vitro *[5,6]. Upon invasion by *C. jejuni*, human epithelial cells respond by secreting cytokines, such as IL-8, which stimulate recruitment of inflammatory cells [2], including macrophages and dendritic cells that engulf and rapidly kill *C. jejuni *[7]. Adherence to epithelial cells is a prerequisite for invasion, and capsular polysaccharides, motility, and a number of surface associated proteins including CadF, CapA, JlpA and FlpA are required for efficient adherence of *C. jejuni *to epithelial cells [8-14]. Furthermore, metabolic processes in *C. jejuni *are also important for invasion of epithelial cells [15,16].

HtrA is a highly conserved periplasmic protein that possesses both protease and chaperone activity [17,18], and it has been demonstrated that HtrA is important for virulence of a number of bacterial pathogens such as *Salmonella enterica *serovar Typhimurium [19], *Listeria monocytogenes *[20], *Klebsiella pneumonia *[21] and *Yersinia enterocolitica *[22]. It is well established that HtrA is important for stress tolerance and survival of most bacteria, because HtrA degrades and prevents aggregation of periplasmic proteins that misfold during stress [23-25], however, only a few studies have investigated the individual role of the protease and chaperone activity of HtrA in virulence. Recently, it was shown that *Salmonella *Typhimurium requires both the HtrA protease and chaperone activity to grow in the liver and spleen of infected mice [26]. In contrast, only the chaperone activity of HtrA is important for spread of *Shigella flexneri *in cultured epithelial cells, possibly because HtrA is involved in the folding of the surface located virulence factor, IcsA [27,28]. This is consistent with a model for outer membrane biogenesis in non-pathogenic *Escherichia coli *proposing that the chaperone activity of HtrA ensures a safe transit of proteins across the periplasm and their assembly into the outer membrane [29]. Thus, even though HtrA is a conserved protein it is unpredictable whether the protease or chaperone activity is involved in virulence.

Several studies have suggested that HtrA is important for virulence of *C. jejuni*. Recently, an insect infection model was used to show that the outcome of a *C. jejuni *infection is affected by HtrA, as fewer *Galleria mellonella *larvae are killed by an *htrA *mutant than by the isogenic wild type strain [30]. In addition, we previously showed that attachment of *C*. *jejuni *to epithelial host cells is highly dependent on HtrA [31]. Furthermore, an *htrA *mutant was isolated from a *C. jejuni *transposon library screened for reduced invasion ability [16]. While several studies have revealed a role of HtrA in virulence of pathogenic bacteria, the question whether HtrA employs the chaperone or protease activity to promote bacterial virulence has received only limited attention. In the present study, we explore the role of HtrA in the virulence of *C. jejuni *by assessing the requirement for each HtrA activity in the interaction with host cells.

## Results and discussion

### Interaction of *C. jejuni htrA *mutants with epithelial cells and macrophages

**Figure 1:**
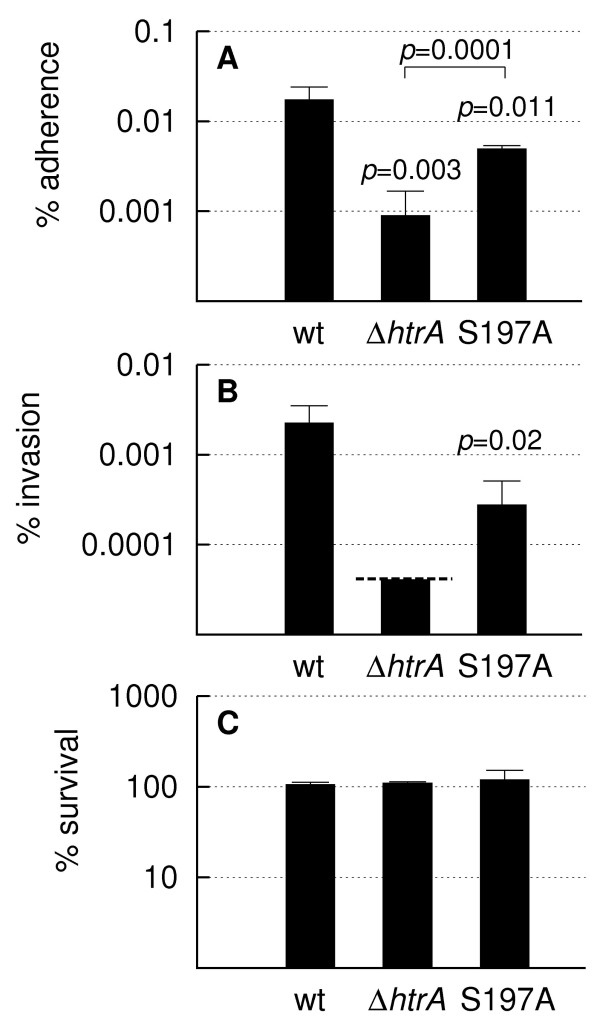
**Effect of mutations in the *htrA *gene of *C. jejuni *on adherence to and invasion of INT407 human intestinal epithelial cells**. Adhered (A), invaded (B), and viable (C) bacteria of *C. jejuni *NCTC11168 (wt), LB1281 (*ΔhtrA*) and KB1025 (*htrA *S197A) expressed as percentage of the inocula. Values represent the average and standard deviation of results from four wells in two independent experiments. Dashed line show the limit of detection for the *ΔhtrA *mutant. The *p *values are calculated using Student's *t*-test. *p *values are obtained by comparing each mutant to the wild type. In Panel A, the two mutants are also compared as indicated in the figure.

HtrA of *C. jejuni *is a dual functional protein possessing both protease and chaperone activity [18]. To investigate which of these activities is important for the interaction of *C. jejuni *with human epithelial cells, we used a mutant (*htrA *S197A) encoding an HtrA protein with a single amino-acid change in the proteolytic site causing the protein to lack protease activity, but retain chaperone activity [18]. The adherence and invasion ability of the *htrA *S197A mutant was compared to the wild type as well as a *ΔhtrA *mutant in a gentamicin protection assay with cultured INT407 epithelial cells. Adherence of the *htrA *S197A mutant was reduced 3-fold compared to the wild type, while adherence of the *ΔhtrA *mutant was reduced 20-fold as reported previously (Figure 1A) [31]. Therefore, both chaperone and protease activity of HtrA are required for optimal adherence, but the chaperone activity alone greatly stimulates adherence. Likewise, invasion of INT407 cells by the *htrA *S197A mutant was reduced 7-fold compared to the wild type, while invasion by the *ΔhtrA *mutant was reduced at least 50-fold as previously reported (Figure 1B) [31]. Adherence is a prerequisite for invasion and the invasive potential can therefore be expressed as the percentage of adherent bacteria that are internalized. This value thus normalizes for variable adherence, and reflects the capacity of already adhered bacteria to invade [32]. We found that the capacity to invade was 13% for the wild type, 6% for the *htrA *S197A mutant, and < 5% for the *ΔhtrA *mutant. Therefore, once the bacteria have adhered to the host cells, *C. jejuni *needs HtrA protease activity for optimal internalization; the exact effect of the chaperone activity on internalization could not be assessed from these results, since no internalized *ΔhtrA *mutants could be recovered. Taken together, the results show that the chaperone activity of HtrA plays a significant role in attachment of *C. jejuni *to host cells. As HtrA is important for stress tolerance in *C. jejuni *[18,31] we tested if the reduced binding of an *htrA *mutant to epithelial cells was simply the result of decreased survival. However, no loss of viability was observed for neither the wild type nor the *htrA *mutants in a control assay (Figure 1C), confirming that the number of CFU measured in the assay reflects the adherence and invasion capabilities of *C*. *jejuni*. This result indicates that the role of HtrA in host cell binding is more specific than improving the general stress response of *C. jejuni*, which is consistent with the observation that reduced host cell binding is not a general feature of *C. jejuni *mutants lacking key stress response proteins, such as ClpP and Lon [33]. Our results also show that the protease activity, and to some degree the chaperone activity, is required for optimal internalization of epithelial cells, although the effect of HtrA on this process is much smaller than the effect on adherence [31]. Reduced invasion by *C. jejuni *of epithelial cells is also observed in the absence of two cytoplasmic proteases, ClpP and Lon [33], indicating that proteolytic activity in general may be important during the process of host cell internalization.

To determine if the loss of HtrA also affects the ability of *C. jejuni *to be taken up by macrophages, cultured J774.1 mouse macrophages were infected with the wild type, the *ΔhtrA*, and the *htrA *S197A mutant, and adherence and phagocytosis were quantified by a gentamicin protection assay. The number of adhered wild-type bacteria ranged from 0.03%-1.2% of the inoculum, while the number of phagocytosed wild-type bacteria ranged from 0.01%-0.19% of the inoculum. To assess the importance of HtrA in these processes the data for each *htrA *mutant was normalized to the wild type (Table 1). The complete lack of HtrA caused a mean reduction in adherence to macrophages of approximately three-fold, while adherence of the *htrA *S197A mutant was not different from that of the wild type (Table 1). Correspondingly, uptake by macrophages of the *ΔhtrA *mutant was reduced five-fold compared to the wild type, while uptake of the *htrA *S197A mutant was not reduced (Table 1). From these data, we conclude that HtrA chaperone activity is important for macrophages to bind *C. jejuni*, and that HtrA does not affect phagocytosis by macrophages once the bacteria have been bound. In addition, the protease activity of HtrA is not important for the interaction of *C. jejuni *with macrophages.

**Table 1:** Interaction of *C. jejuni htrA *mutants with macrophages

Strain	Adherence*^a^*	Phagocytosis*^a^*
Wild type	100	100

LB1281 (*ΔhtrA*)	30 ± 10	21 ± 9

KB1025 (*htrA *S197A)	96 ± 33	93 ± 44

*^a^*Percentage of the value for the wild type ± standard error.

Taken together, these results show that HtrA chaperone activity plays an important role in the attachment of *C. jejuni *to both epithelial cells and macrophages. We previously showed that the chaperone activity of HtrA is important for periplasmic protein homeostasis in *C. jejuni *under non-stress conditions [18], and we therefore speculate that HtrA of *C. jejuni *is involved in folding of periplasmic or outer membrane proteins. In *E. coli *and *Shigella*, HtrA acts as a chaperone that mediates proper folding and insertion of proteins into the outer membrane [27,29]. In *C. jejuni*, HtrA may therefore be essential for the function of one or several adherence factors, which may explain why lack of HtrA has a 5-10 times larger effect on adherence than lack of any single surface adhesin, such as CadF, CapA, JlpA and FlpA [11-14]. Interestingly, *C. jejuni *lacking PEB4, a homolog of the periplasmic chaperone SurA, adheres to epithelial cells 50-100 fold less efficiently than wild-type [34], emphasizing the importance of periplasmic chaperones for the adherence of *C. jejuni *to host cells.

### Surface properties and protein secretion of *C. jejuni htrA *mutants

**Figure 2:**
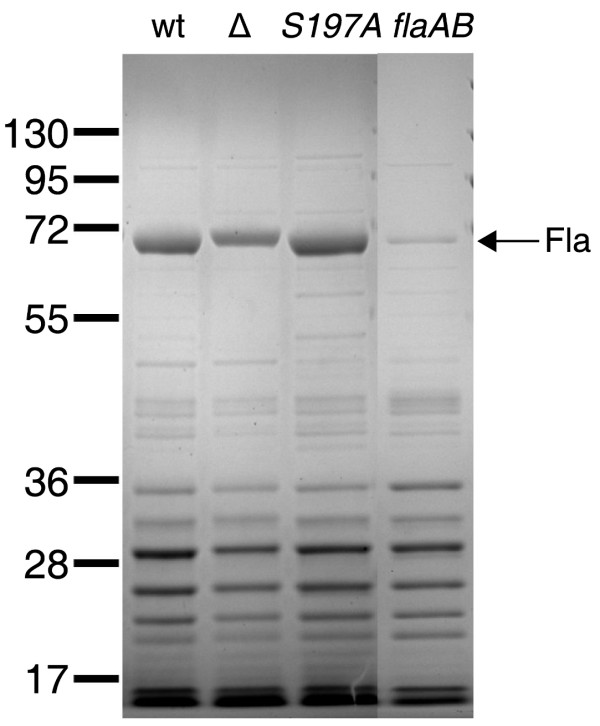
**Effect of mutations in the *htrA *and the *flaAB *genes of *C. jejuni *on the composition of surface proteins**. Glycine extracts from *C. jejuni *NCTC11168 (wt), LB1281(*ΔhtrA*) and KB1025 (*htrA *S197A) separated by SDS-PAGE and stained with Coomassie Brillant Blue. The positions of molecular weight standards are indicated on the left (in kilodaltons).

To pin-point the role of HtrA in the *C. jejuni*-cell interaction, we investigated if the surface protein pattern was altered in the *htrA *mutants. We compared the surface protein profiles using glycine-extracts from the wild type, the *ΔhtrA *mutant, and the *htrA *S197A mutant. This method allows extraction of several surface proteins [35-37] known to interact with eukaryotic cells [10,38,39], but we did not identify proteins with an altered abundance in the *htrA *mutants compared to the wild type (Figure 2). However, lack of HtrA in bacteria may cause inactive proteins to appear on the bacterial surface, without affecting the *amount *of the particular protein. In *Shigella*, for example, loss of HtrA does not affect the amount of the virulence factor, IcsA, in the outer membrane, but instead causes IcsA to adopt an altered conformation with reduced activity [27]. Similarly, in the pathogenic *E. coli *O157:H7, it is proposed that the absence of *htrA *causes an autotransporter protein, involved in virulence, to misfold and wrongly insert into the outer membrane [40]. In addition to the adhesins mentioned above, the antigens PEB1 and PEB3 are important for adherence of *C. jejuni *to eukaryotic cells [10,34](B.W. Wren and R. Langdon, pers. comm.). Interestingly, PEB1 isolated from non-adherent *C. jejuni *strains exists in slightly different isoforms compared to PEB1 isolated from adherent strains, indicating that small changes in PEB1 may affect adherence [10]. We speculate that the reduced adherence of the *htrA *mutants to epithelial cells is caused by misfolding or wrong localization of one or more adhesive proteins. Alternatively, HtrA may affect other periplasmic processes such as *N*-linked glycosylation of surface proteins like PEB3 and thus affect adherence to epithelial cells and macrophages [41]. Cells of the innate immune response, such as macrophages, identify infecting bacteria by recognition of a variety of molecular ligands on the bacterial surface, and it is possible that HtrA affects such ligands by similar mechanisms to those considered for the adhesins.

**Figure 3:**
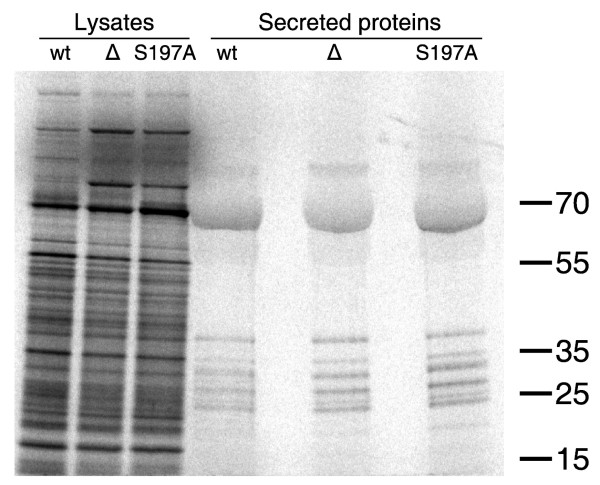
**Effect of mutations in the *htrA *gene of *C. jejuni *on secretion of Cia proteins**. Secretion of Cia proteins were induced by growing *C. jejuni *NCTC11168 (wt), LB1281(*ΔhtrA*) and KB1025 (*htrA *S197A) in the presence of sodium deoxycholate followed by radioactive labelling in RPMI medium containing FBS. Labeled proteins in the bacterial lysates and in the supernatants were separated by SDS-PAGE and detected by autoradiography. The positions of molecular weight standards are indicated on the right (in kilodaltons).

We observed a slight difference in mobility of FlaA in the *ΔhtrA *mutant compared to the wild type and the *htrA *S197A mutant, which correlated with our detection of a single nucleotide insertion in the homopolymeric tract (nucleotides 1,227,121 to 1,227,129 in reference genome NCTC11168) of gene *cj1295 *in the two latter strains, resulting in a premature stop codon. A similar polymorphism in *cj1295 *have previously been shown to cause a mobility shift of FlaA [42]. Bacterial motility, however, did not vary between the strains, nor did the appearance of the flagellum by electron microscopy [31]. Upon contact with human epithelial cells, *C. jejuni *secretes a set of proteins through the flagellum called *Campylobacter *invasive antigens (Cia), which have been shown to be important for invasion [43,44]. We tested if loss of *htrA *affects the secretion of Cia proteins by inducing Cia expression and secretion with sodium deoxycholate and bovine calf serum as described previously [45]. However, the protein pattern in the supernatant was not affected by lack of HtrA (Figure 3), and the reduced interaction with epithelial cells of *C. jejuni *mutants lacking HtrA is therefore not likely to be caused by impaired secretion of the these virulence factors.

## Conclusion

It has long been known that HtrA is important for virulence of pathogenic bacteria, but not much attention has been given to the role of the individual activities of HtrA. This study demonstrates that particularly the chaperone activity of HtrA has a significant impact on the interaction between *C. jejuni *and host cells. Lack of HtrA reduced bacterial binding to epithelial cells 5-10 times more than lack of any known adhesin [11-14], suggesting a pleiotropic effect. Even though HtrA traditionally has been viewed as a stress response protein, our data indicate that HtrA has specific functions during infection that may be stress-independent. This suggestion correlates with growing evidence from other bacteria showing that the chaperone activity of HtrA is involved in folding of virulence factors.

## Methods

### Bacterial strains and growth conditions

*C. jejuni *NCTC11168 (National Collection of Type Cultures), *C. jejuni *NCTC11168 *htrA::cat *(LB1281, [31]), *C. jejuni *NCTC11168 *htrA *S197A (KB1025, [18]), and *C. jejuni *NCTC11168 *flaAB *(CV1178) were routinely grown on blood agar base II (Oxoid) supplemented with 5% bovine blood, or in brain heart infusion (BHI) broth (Oxoid) at 37°C in a microaerobic environment (6% O_2_, 6% CO_2_, 4% H_2_, and 84% N_2_). CV1178 was constructed by natural transformation of *C. jejuni *NCTC11168 with chromosomal DNA from a *C. jejuni *81116 *flaAB *mutant [46], followed by selection for kanamycin resistance.

### Cell cultures

Stock cultures of INT 407 human embryonic intestinal epithelial cells were grown in minimal essential medium (MEM; Gibco) supplemented with 10% (vol/vol) fetal bovine serum (FBS; Gibco) and maintained at 37°C in a humidified, 5% CO_2 _incubator. Stock cultures of J774.1 murine macrophage-like cells were grown in RPMI1640 (Gibco) supplemented with 10% (vol/vol) FBS and maintained at 37°C in a humidified, 5% CO_2 _incubator.

### Gentamicin protection assay

Adherence and invasion/phagocytosis assays were performed with monolayers of INT407 epithelial cells or J774.1 macrophage cells growing in MEM (INT407) or RPMI (J774.1) supplemented with 10% FBS at 37°C in a humidified microaerobic atmosphere containing 5% CO_2_. Approximately 4 × 10^7 ^bacterial cells in MEM or RPMI were centrifuged at 400 rpm onto a monolayer consisting of 4 × 10^5 ^host cells and incubated for 1 h. The actual inocula were enumerated by plate count. To determine adherence, the monolayers were washed three times with 0.9% NaCl, and host cells were lysed by adding 0.1% Triton X-100. Adhered bacteria were enumerated by plate count. To determine invasion/phagocytosis, the infected monolayers were incubated in MEM or RPMI containing 100 μg ml^-1 ^gentamicin for 2 h at 37°C microaerobic-5% CO_2 _atmosphere to kill extracellular bacteria. The monolayers were washed three times with 0.9% NaCl, host cells were lysed with 0.1% Triton X-100, and internalized bacteria were enumerated by plate count. For the macrophage assay, the data from four individual experiments in duplicate was normalized to the values for the wild type, due to large variations between individual experiments. For the viability-control assay, a monolayer consisting of 4 × 10^5 ^INT407 cells was incubated in 1 ml MEM supplemented with 10% FBS at 37°C in 5% CO_2_. After 1 h, 900 μl medium was transferred to an empty well and approx. 4 × 10^7 ^bacterial cells were added and incubated for 1 h at 37°C in a humidified microaerobic atmosphere containing 5% CO_2_. Subsequently, Triton X-100 was added to 0.1% and bacteria enumerated by plate count.

### Protein secretion

Secreted *C. jejuni *proteins were detected essentially as described in [45] and [43] with minor modifications. Briefly, *C. jejuni *NCTC11168, LB1281 and KB1025 were cultured O/N in Mueller-Hinton (MH) broth on MH agar containing 0.1% sodium deoxycholate (Sigma) to stimulate expression of the *cia *genes [45]. Bacteria were harvested in RPMI1640 without methionine (R7513, Sigma), pelleted at 8000 × *g*, and washed twice in RPMI1640. Six milliliters of a bacterial suspension containing approximately 10^9 ^CFU was labeled with L-^35^S-methionine (Perkin Elmer Life Sciences) at a concentration of 50 μCi ml^-1^, and incorporation of ^35^S-methionine was allowed for 30 min at 37°C under microaerobic conditions. Subsequently, the bacterial suspensions were incubated microaerobically at 37°C for 30 min with 128 μg ml^-1 ^chloramphenicol to stop protein synthesis. The suspensions were then incubated with 1% FBS (Gibco) for 30 min at 37°C under microaerobic conditions to stimulate protein secretion. Subsequently, the bacteria were harvested at 8000 × *g*, and the supernatants, containing the secreted proteins, were filtrated through a 0.2 μm filter, and proteins were concentrated by adding five volumes of ice-cold 1 mM HCl-acetone followed by incubation O/N at -20°C. The precipitated proteins were sedimented at 13,000 × *g *and resuspended in water, followed by dialysis (6-8000 MWCO) to remove un-incorporated ^35^S-methionine. The secreted proteins and whole-cell lysates were separated by SDS-PAGE and the dried gels were exposed to a phosphorimager screen (PerkinElmer) to detect labeled proteins.

### Surface proteins

Surface associated proteins were extracted essentially as described by McCoy et al. [36]. Briefly, *C. jejuni *cells were harvested from blood agar plates, and the pellet was resuspended in 0.2 M glycine-HCl pH 2.2 and incubated at room temperature for 10 min followed by removal of cells at 6,000 × *g*. Protein content in the extracts was quantified by densitometri of Amido Black stained proteins with BSA as standard, and equal amounts were separated by SDS-PAGE and stained with Coomassie Brillant Blue.

## Competing interests

The authors declare that they have no competing interests.

## Authors' contributions

KTB participated in the design of the study, performed experiments, conducted data analysis, and drafted the manuscript. CSV and LB participated in the design of the study and edited the manuscript. All authors read and approved the final manuscript.
